# Transfusion-transmitted infections

**DOI:** 10.1007/s44313-024-00014-w

**Published:** 2024-04-12

**Authors:** Han Joo Kim, Dae-Hyun Ko

**Affiliations:** Department of Laboratory Medicine, Asan Medical Center, University of Ulsan College of Medicine, 88 Olympic-Ro, Songpa-Gu, Seoul, 05505 Korea

**Keywords:** Transfusion-transmitted infection, Hepatitis B virus, Hepatitis C virus, Human immunodeficiency virus, Pathogen inactivation

## Abstract

The risk of transfusion-transmitted infection (TTI) has always existed because transfused blood products are biological materials derived from humans. To prevent TTIs, screening strategies have been developed for various infectious diseases, such as hepatitis B virus, hepatitis C virus, and human immunodeficiency virus, contributing significantly to reducing TTI globally. Nevertheless, septic transfusion reactions (STRs) due to bacterial contamination remain an unresolved issue. Various infectious diseases can be transmitted through blood products, and preventive and selective screening strategies have been applied across different regions. Although multiple strategies, including culture-based and rapid detection kit-based methods, have been introduced to overcome STRs, complete prevention has not yet been achieved. Recently, pathogen inactivation methods have been developed to eliminate non-specific organisms rather than screening specific organisms. This approach is anticipated to contribute significantly to diminishing the risk of TTIs in the future.

## Introduction

Due to the nature of human-derived products, transfusion blood products cannot be free from the risk of transmission-transmitted infection (TTI). Although various screening tests are conducted globally to prevent TTI, technological limitations hinder the complete elimination of this risk. This review aims to explore the current status of TTI risk and present insights into future perspectives.

## Transfusion-transmitted infectious agents and blood screening policies

Screening tests are well established for globally significant TTI-related infectious agents. These commonly include screening for hepatitis B virus (HBV), hepatitis C virus (HCV), human immunodeficiency virus (HIV), and syphilis. Moreover, depending on the country, tests for human T-cell lymphotropic virus (HTLV), West Nile virus (WNV), cytomegalovirus, Babesia, and Zika viruses may also carried out. Here, we briefly review each infectious agent.

## Viruses

### HIV

HIV is a pathogen that causes acquired immune deficiency syndrome and was first identified in 1983 [1]. The risk of transmission through transfusion was suggested in a case of *Pneumocystis jirovecii* pneumonia in a patient with hemophilia A treated with a factor VIII concentrate [2]. Furthermore, in the 1980s, reports indicated that over 50% of patients with hemophilia A had HIV antibodies [3]. As these risks became known, the need for HIV screening tests before blood donation became apparent. In the USA, the HIV antibody test was introduced in 1985 as a screening test for blood donations [4]. Since the development of the nucleic acid test (NAT), which shortens the window period to approximately 10 days, many countries have adopted NAT as an HIV screening test. However, some countries still exclusively perform HIV antibody tests based on the assumption that there are minimal additional cases that can be detected with NAT but not antibody testing. Furthermore, given the trend to regard HIV as a controllable disease, there is assumed to be no need to conduct an expensive test universally. In Korea, NAT for HIV detection was introduced in 2005 as a screening test for blood donations [5].

### HBV

HBV was first discovered by Blumberg in 1964 [6]. Before the 1970s, approximately 6% of patients who received multiple transfusions had transfusion-transmitted HBV [7]. HBV tests can be broadly divided into serological and DNA tests. Serological tests detect HBsAg, anti-HBs antibody, and anti-HBc antibody. The United States Food and Drug Administration (FDA) mandates testing for HBsAg, total anti-HBc antibodies, and HBV DNA in blood donations. In Korea, only HBsAg and HBV DNA tests are regularly performed. Korea is classified as an HBV intermediate endemic area, with a higher prevalence of HBV infection than Western countries. Therefore, HBV testing was one of the first screening tests introduced in Korea in the 1970s. Although the national vaccination program has led to a gradual decrease in HBV prevalence, the estimated residual risk remains higher than that in Western countries [5, 8].

### HCV

Before the identification of HCV, post-transfusion hepatitis non-A, non-B (PTH-NANB) was reported in over 10% of patients who had received blood transfusions. However, with the identification of HCV in 1989 and the introduction of the HCV antibody test, transfusion-transmitted HCV incidence sharply decreased [9, 10]. The HCV antibody test was introduced as a blood-donation screening test in the USA in 1990 and in Korea in 1991. Subsequently, in 2005, the HCV NAT was introduced to further reduce the risk, and is now rapidly spreading to many other countries [11]. In Korea, the residual risk of transfusion-transmitted HCV infection is approximately 0.27 per 10^6^ transfusions [5]. A distinctive aspect of blood donor screening in Korea is the alanine transaminase (ALT) test, which is not performed in Western countries [12]. ALT tests have long been used to detect PTH-NANB. However, with the discovery of HCV infection and the development of new test methods, ALT tests are no longer routinely performed in most countries. In the USA for example, ALT testing was excluded from blood donor screening in the 2000s. In Korea, despite changing the criteria for inappropriate blood from 65 to 101 IU/mL, the ALT test is still included in blood donation screening. Nevertheless, to prevent unnecessary disposal of limited blood resources, in-depth discussions are needed to determine whether ALT testing is still required.

### Other viruses

WNV is an ssRNA virus in the Flaviviridae family that causes a vector-borne disease. Due to its geographical distribution, WNV has received relatively less attention in Asia. However, in North America and Europe, it is a clinically important infectious agent, and TTI cases have long been reported [13]. NAT is more effective for preventing WNV TTI than serological tests. Therefore, in many Western countries, WNV NAT has been widely used for screening blood donations, leading to the development and utilization of numerous commercial platforms [4, 14].

The Zika virus, another vector-borne pathogen, typically causes asymptomatic infection in humans; however, it can occasionally lead to severe disease, resulting in fatal outcomes for fetuses. Although Zika virus is typically transmitted by mosquitoes, TTIs have also been reported. In a study in Brazil, 0.16% of blood donors tested positive for Zika virus RNA [15]. Methods such as NAT and pathogen inactivation (PI) are effective for preventing Zika virus TTI [16]. In Korea, there is currently no regular screening for WNV or Zika virus.

HTLV is a lipid-enveloped RNA retrovirus that causes adult T-cell leukemia/lymphoma in humans. Infection routes include vertical transmission, sexual contact, and transfusion. Transfusion-related transmission is usually associated with blood cell components. In Korea, blood donation screening has included anti-HTLV antibody testing since 2008, covering all donated blood samples. The prevalence of anti-HTLV antibody positivity among donated blood samples in Korea is 0.027% [17]. As HTLV is found within white blood cells, implementing leukoreduction can reduce transmission risk [4].

## Bacteria

Blood for transfusions is typically collected under aseptic conditions and is generally considered sterile, with no bacteria present; however, bacterial contamination has long been documented. These bacteria are generally transferred from the normal skin flora because of insufficient disinfection. The bacterial contamination rate can be effectively reduced by attaching a diversion bag to the blood collection bag. This prevents the initial blood drawn from mixing with blood intended for transfusion [18]. However, bacterial contamination of blood products remains an issue. In particular, platelet products stored at room temperature are vulnerable to bacterial contamination, and between 1 in 1000 and 1 in 2500 units of platelets are bacterially contaminated. As a result, the prevalence of septic transfusion reactions (STRs) in hematology or oncology patients who receive large amounts of platelet products is estimated to be as low as 1 in 1000 [19]. Transfusion-transmitted sepsis has been identified and confirmed through culture in at least one in 100,000 recipients.

Various measures have been implemented to prevent STRs caused by bacterial contamination. Broadly, these methods can be classified into two categories: culturing platelet products and using rapid detection kits. In the former category, BacT/ALERT® (BioMérieux, Marcy-l’Étoile, France) has received FDA approval for use in platelet quality control purposes, while in the latter category, commercial kits, such as BacTx® assay (Immunetics, Marlborough, MA, USA) and the Platelet Pan Genera Detection Test (Verax Biomedical, Marlborough, MA, USA) are available. Although there are variations among countries in the adoption of these methods, no method has completely prevented STRs caused by bacterial contamination. In Korea, full-scale testing for bacterial contamination has not yet been implemented, and testing is limited to a subset of samples from blood centers.

## Other agents

In addition to the aforementioned infectious agents, various diseases such as *Treponema pallidum*, *Trypanosoma cruzi*, Babesia, and malaria can be transmitted through blood transfusion. Prevention and screening strategies for each case are applied on a national or regional basis.

## PI

Strategies for screening to prevent TTI continue to evolve. However, all tests have inherent limitations for detecting early infections. Furthermore, with the identification of new infectious diseases, there are limitations to preventing all TTIs through screening alone. Therefore, PI has recently been adopted by many countries.

PI methods have been developed for each blood product and vary according to the intended use. Plasma products are analyzed using solvent/detergent methods (Octapharma, Lachen, Switzerland) and methylene blue methods (Macopharma, Mouvaux, France). Platelet product methods include adding amotosalen (Cerus Corporation, Concord, CA, USA) or riboflavin (Terumo BCT, Inc., Lakewood, CO, USA) and exposure to UV light to destroy DNA or RNA structures [20–23]. These methods have various advantages and disadvantages, and each method is applicable to different blood products, with different target infectious agents and cost disparities. When adopting PI, blood product quality must not be compromised and the associated costs should be considered. Currently, these methods are widely adopted in Europe but have not been introduced in Korea [24–26].

## Conclusions

For decades, global efforts have been made to reduce TTI and have successfully mitigated the risk of many infectious diseases. However, complete elimination of TTI has not yet been achieved. Therefore, efforts to enhance blood safety should continue in the future.

## Data Availability

No datasets were generated or analysed during the current study.
